# Case Report: A Case of Unusual Combination of Hypothyroidism, Myocardial Bridging, and Myocardial Infarction-Induced Left Ventricular Aneurysm

**DOI:** 10.3389/fcvm.2021.684616

**Published:** 2021-11-15

**Authors:** Yueliang Li, Zhengjiang Liu

**Affiliations:** Department of Cardiology, Qingyuan People's Hospital, the Sixth Affiliated Hospital of Guangzhou Medical University, Qingyuan, China

**Keywords:** myocardial bridging, myocardial infarction, ventricular aneurysm, percutaneous coronary, hypothyroidism

## Abstract

**Background:** Myocardial bridging (MB) of the coronary artery is a congenital anatomical variation, which has traditionally been considered a benign condition that does not cause cardiovascular events. However, recent studies have shown that MB is associated with major adverse cardiac events, including angina, myocardial infarction, arrhythmia, syncope, and even sudden death.

**Case:** We report a case of a 41-year-old man who had hypothyroidism and MB associated with ventricular aneurysm following myocardial infarction. This patient was admitted to our hospital because of 11 days of sudden discomfort and pain in the chest. An electrocardiogram on admission showed an old myocardial infarction. Coronary angiography showed MB in the distal segment of the left anterior descending artery. Left ventricular angiography, which was performed using a pigtail catheter, showed ventricular aneurysm formation. Thyroid ultrasound demonstrated hypothyroidism and Hashimoto's thyroiditis. Patients with hypothyroidism and MB have a high risk of acute myocardial infarction or even sudden death.

**Conclusion:** Observations in our case suggest that early recognition of hypothyroidism and MB is important for risk stratification and prognosis in patients with myocardial necrosis and acute coronary syndrome. Additionally, this early recognition may have positive effects on cardiovascular outcomes in patients with hypothyroidism.

## Introduction

Myocardial bridging (MB) of the coronary artery is a congenital coronary anomaly, which has traditionally been considered a benign condition. The prevalence of MB varies greatly owing to different methods and criteria used for detection, with a much higher prevalence by intravascular ultrasound or autopsy than by angiography (1). MB is most commonly localized in the middle segment of the left anterior descending artery (LAD). Although the intramural portion is usually preserved, atherosclerotic plaques are frequently observed in the segment proximal to MB (2). MB results in compression of the coronary artery during systole, which can cause angina pectoris, myocardial infarction, left ventricular dysfunction, paroxysmal atrioventricular block, effort-related myocardial ischemia, and sudden cardiac death (3, 4). Ishikawa et al. reported pathological and anatomical evidence of MB and showed that MB was a risk factor for coronary atherosclerosis and myocardial infarction (5).

Ventricular aneurysm is a common complication after acute myocardial infarction. A large area of myocardial infarction and reduced or loss of local contractile force can result in left ventricular remodeling and even severe conditions, such as myocardial fibrosis, cardiac cavity dilatation, and increased ventricular wall tension. This situation then causes the ventricular wall to bulge outwards, leading to the formation of ventricular aneurysm. Ventricular aneurysm is characterized by saclike or irregular out-pouching of the heart, a thinner regional ventricular wall, and no or paradoxical wall motion. Ventricular aneurysm causes left ventricular dysfunction, arrhythmia, and mural thrombus formation and increases the risk of cardiovascular events in patients with acute myocardial infarction (6).

We present here a case of a 41-year-old man with an unusual combination of hypothyroidism caused by Hashimoto's thyroiditis, MB, and myocardial infarction-induced ventricular aneurysm. Written informed consent was obtained from the patient for publication of this case report.

## Case Description

**Figure 1:**
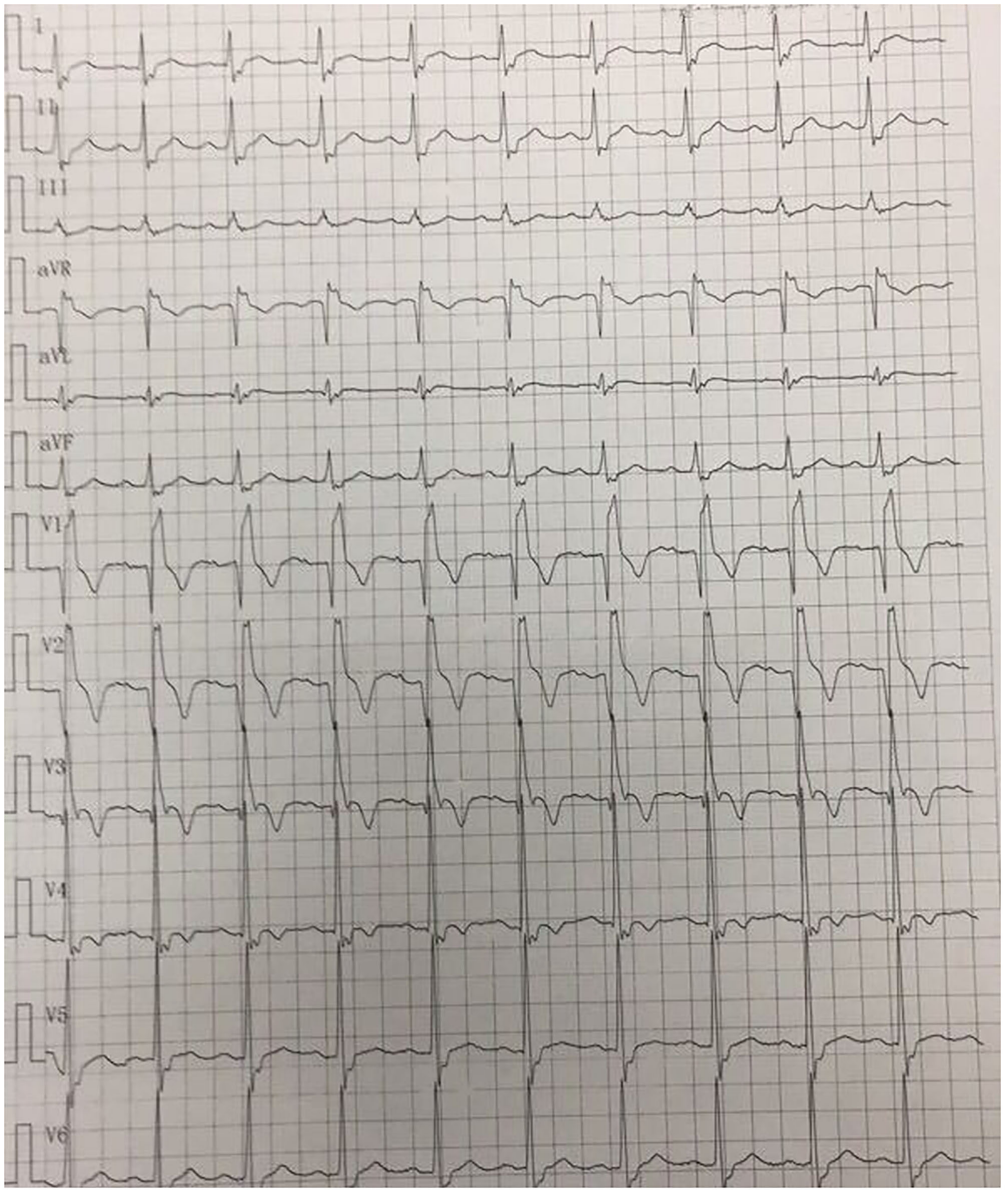
Electrocardiogram on admission shows sinus rhythm, complete right bundle branch block, pathological Q waves in leads V1–V3, and T wave changes in some leads.

**Figure 2:**
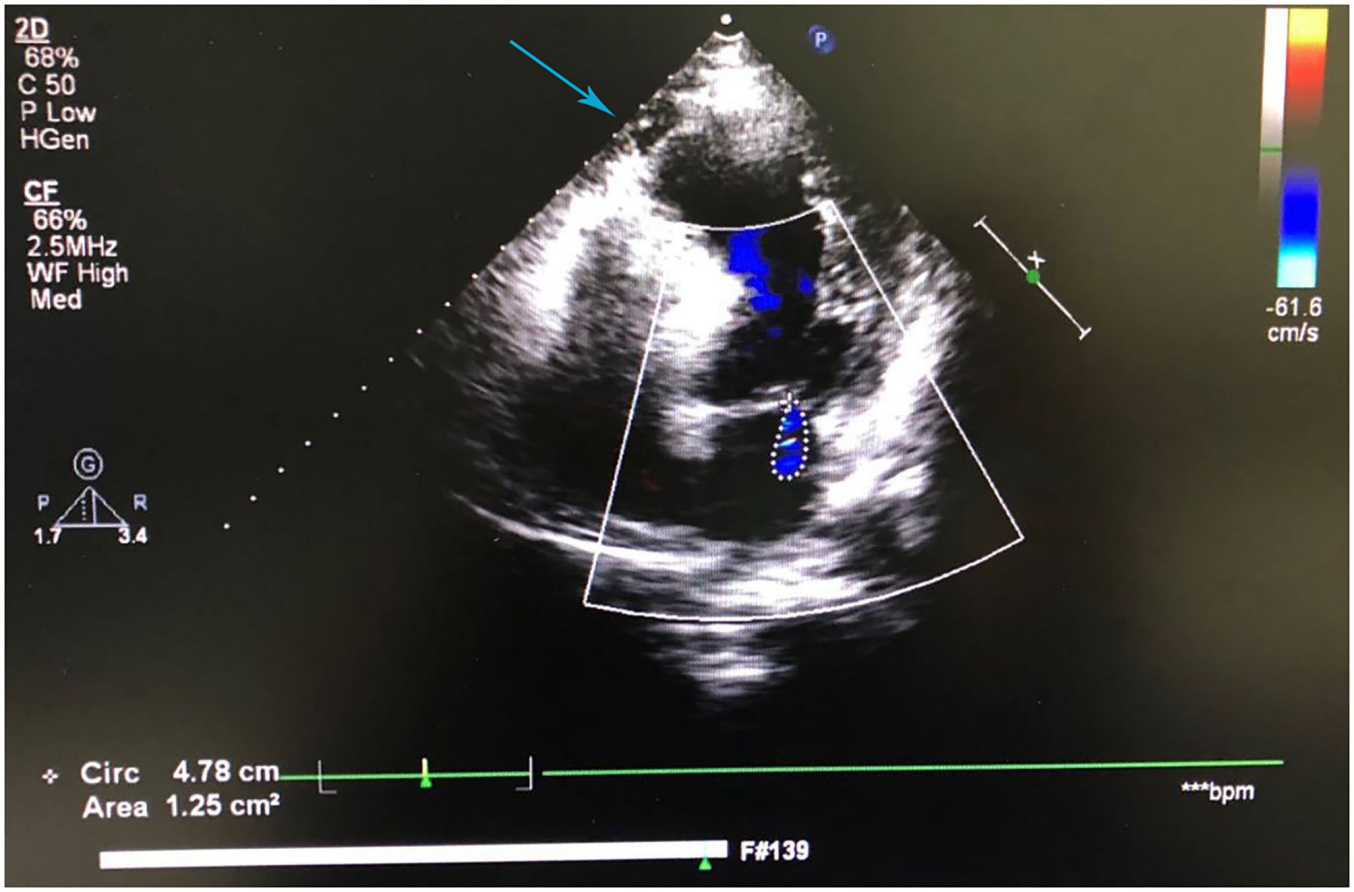
Color Doppler echocardiography shows left ventricular apical aneurysm formation and left ventricular systolic and diastolic dysfunction.

**Figure 3:**
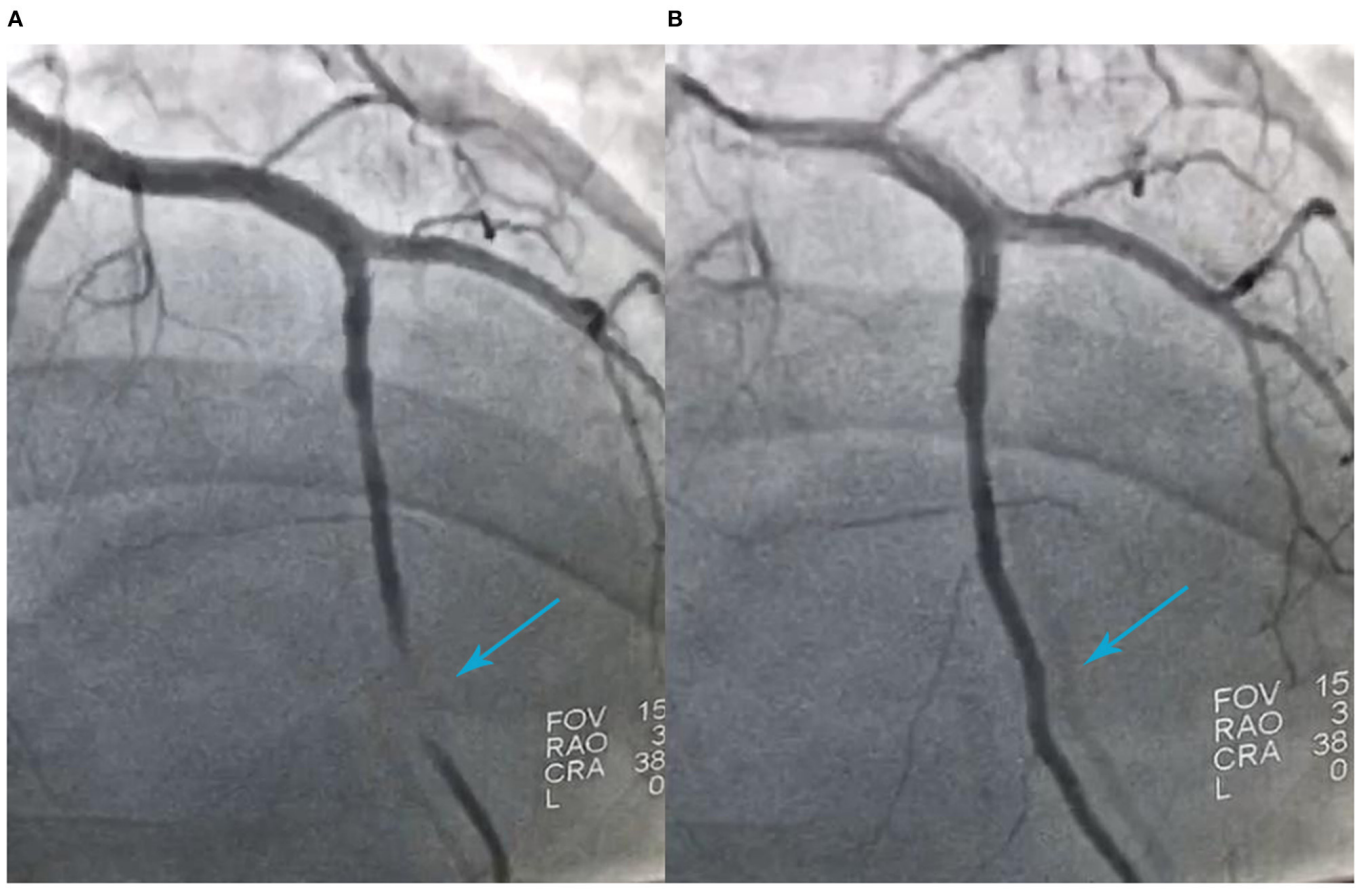
Coronary angiography shows 40%−50% stenosis in the proximal segment of the left anterior descending artery (LAD), myocardial bridging in the distal segment of the LAD during ventricular systole **(A)**, filling of the distal LAD during ventricular diastole **(B)**, and forward flow of TIMI grade 3.

**Figure 4:**
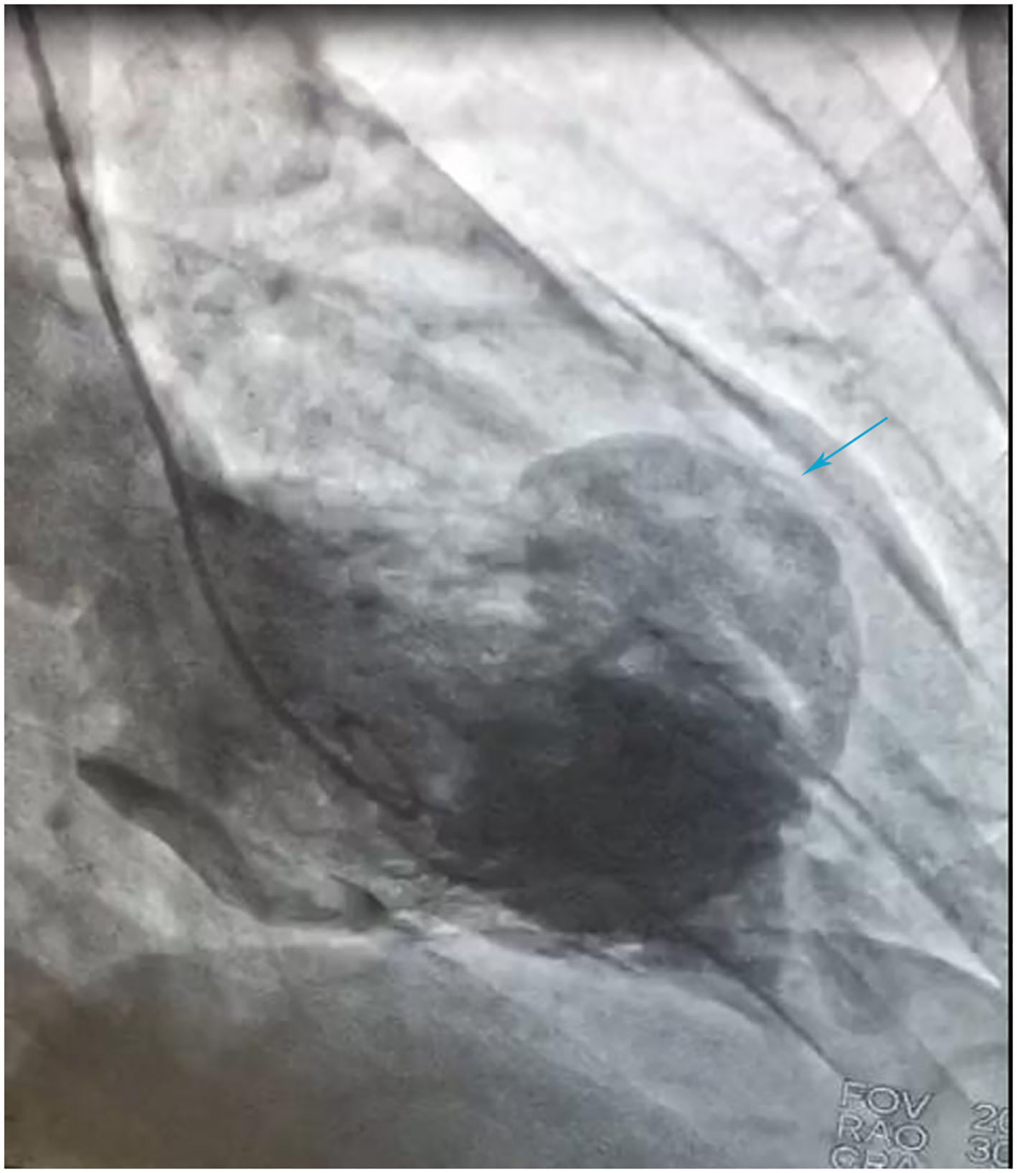
Left ventricular angiography using a pigtail catheter shows ventricular aneurysm formation.

A 41-year-old man was admitted to our hospital because of 11 days of sudden discomfort and pain in the chest. Approximately 3 months previously, the patient experienced chest tightness and discomfort, lasting for approximately 5 min, during exercise, excitement, and emotional upset. These symptoms were relieved with rest. He also experienced fatigue and coldness. Eleven days before admission, he had sudden onset of chest tightness and crushing pain, which persisted without relief and did not radiate to the surrounding areas. On admission, the patient showed a dull facial expression and minimal attentiveness and concentration, and his skin was rough. A physical examination showed that his vital signs were stable. His blood pressure was 120/70 mmHg, heart rate was 85 beats/min with regular rhythm, and no pathological murmurs or pericardial friction rubs were heard over each valve area after auscultation. No obvious abnormal signs were observed in the lungs or abdomen, and there was no pitting edema in the bilateral lower extremities. He denied a previous history of hypertension or diabetes. An electrocardiogram performed on admission showed sinus rhythm, complete right bundle branch block, pathological Q waves in leads V1–V3, and T wave changes in some leads (Figure 1). Color Doppler echocardiography revealed abnormal echoes in the mid and apical segments of the left ventricular anterior wall and segmental wall motion abnormalities, which were consistent with ultrasound features of coronary heart disease. The apex of the left ventricle was dilated at the right ventricular apex. The ventricular wall was thin and the thinnest area was 17 mm. The ventricular wall bulged outwards and downwards and it showed paradoxical movements. Mural thrombus was not observed, and a left ventricular apical aneurysm had formed. The left ventricular ejection fraction was 36%. Echocardiography showed left ventricular systolic and diastolic dysfunction (Figure 2). Thyroid function tests showed the following: triiodothyronine (T3) level, 0.504 nmol/L; free triiodothyronine (FT3) level, 1.66 pmol/L; thyroxine (T4) level, 12.35 nmol/L; free thyroxine (FT4) level, 1.91 pmol/L; thyrotropin (TSH) level, 168.531 mIU/L; antithyroglobulin antibody (TgAb) level, 69.9I U/ml; thyroid peroxidase antibody (TPOAb) level, 486.9 IU/ml; total cholesterol (TC) level, 6.08 mmol/L; triglycerides (TG) level, 5.87 mmol/L; high-density lipoprotein cholesterol (LDLC) level, 0.97 mmol/L; low-density lipoprotein cholesterol (LDLC) level, 4.36 mmol/L; apolipoprotein A (apoA) level, 1.08 g/L; apolipoprotein B (apoB) level, 2.53 g/L; prothrombin time (PT), 14.9 s; international normalized ratio (INR), 1.14; activated partial thromboplastin time (aPTT), 57.8 s; thrombin time (TT), 111.2 s; fibrinogen (FIB) level, 3.23 g/L; aspartate aminotransferase (AST) level, 110 U/L; lactate dehydrogenase (LDH) level, 251 U/L; creatine kinase (CK) level, 169 U/L; creatine kinase-MB (CKMB) level, 10 U/L; hydroxybutyrate hydrogenase (HBD) level, 128 U/L; and troponin T level, 12.3 pg/ml. Ultrasound of the thyroid showed Hashimoto's thyroiditis. Coronary angiography showed that there was no obvious stenosis in the left main coronary artery. Additionally, 40–50% stenosis was found in the proximal LAD, plaques and MB were observed in the distal LAD, and forward flow was TIMI grade 3. There was no obvious stenosis in the left circumflex coronary artery or right coronary artery, and forward flow was TIMI grade 3 (Figures 3A,B). Left ventricular angiography, which was performed using a pigtail catheter, showed formation of ventricular aneurysm (Figure 4).

The abovementioned findings suggested the diagnosis of coronary atherosclerotic heart disease, old myocardial infarction, and ventricular aneurysm formation. Acute myocardial infarction was excluded. On the basis of our findings of thyroid function tests showing decreased T3, FT3, T4, and FT4 levels and increased TSH and TPOAb levels, hyperthyroidism was excluded, and the diagnosis of hypothyroidism and Hashimoto's thyroiditis was made.

A total of 75 mg of clopidogrel with 100 mg of Bayaspirin was administered orally once daily to inhibit aggregation of platelets. Additionally, 20 mg of atorvastatin calcium tablets were administered orally once daily for lowering lipid and stabilizing plaques. Furthermore, a 23.7-mg metoprolol succinate extended-release tablet was administered orally once daily to slow down heart rate. The patient also received thyroid hormone replacement therapy (levothyroxine). The starting dose of levothyroxine was 12.5 μg/day with a progressive increase of 25 μg/day every 2 weeks. He was also treated with conservative medical therapy for ventricular aneurysm.

**Figure 5:**
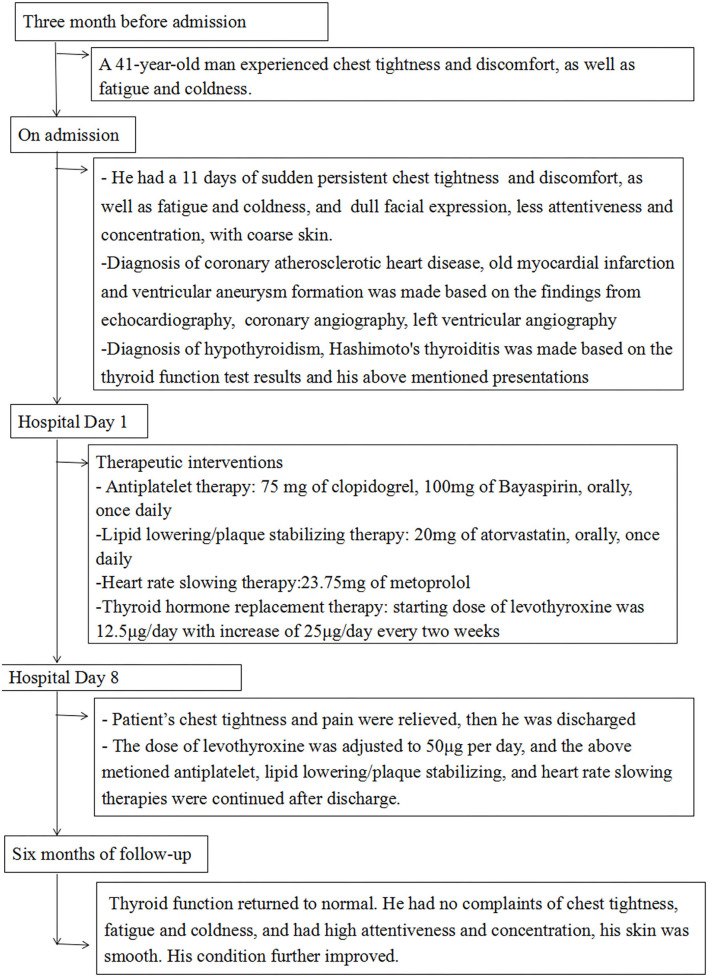
Timeline showing the clinical course in this patient.

After 8 days of treatment, the chest tightness and pain of the patient were relieved, and he was then discharged. The patient was advised to follow a low-sodium, low-fat diet; take medications as prescribed; and exercise appropriately. He was not advised to consume foods containing iodine. He was asked to return to the clinic regularly every month to test thyroid function and to have echocardiography and thyroid ultrasound performed. The dose of levothyroxine was adjusted to 50 μg/day. Additionally, oral administration of clopidogrel (75 mg/day), Bayaspirin (100 mg/day), atorvastatin calcium tablets (20 mg/day), and metoprolol succinate extended-release tablets (23.75 mg/day) was continued. After 6 months of follow-up, thyroid function returned to normal. There was no complaint of chest tightness, fatigue, or coldness, and his skin was smooth and thin. He provided correct answers to asked questions, he had high attentiveness and concentration, and his condition further improved (Figure 5). The patient refused to receive further cardiac surgery for left ventricular aneurysm.

## Discussion

Chest pain is the most common reason for visiting a doctor for patients with MB, most commonly occurs during exertion or exercise, and may also occur at night during sleep and during emotional stress. In the present case, MB was located in the distal segment of the LAD and was not found in the middle segment, and it caused chest pain. This led to myocardial infarction and formation of left ventricular apical aneurysm. The mechanisms by which MB causes chest pain include reduced coronary blood flow and abnormal endothelial function, thrombosis, and coronary spasm (7). MB can induce coronary heart disease. The reason for this induction may be that MB causes compression of the artery during systole, resulting in delayed diastolic relaxation, reduced coronary flow reserve, and decreased blood perfusion. Additionally, repeated compression of the arteries in regions near MB during systole can cause endothelial dysfunction and changes in hemodynamics, thus increasing the risk of coronary artery atherosclerotic stenosis.

Ishikawa et al. found that unstable plaque-related lesion characteristics are more common in MB and coronary arteries near MB are more susceptible to rupture, causing myocardial infarction in young people (8). Therefore, MB is considered a new anatomical risk factor for coronary atherosclerosis. MB initiates the development of atherosclerotic lesions and promotes progression of atherosclerosis at its proximal segment. Compression of the mural coronary artery by MB can be relieved and disappear during diastole. However, fixed stenosis may also exist at the proximal segment, which reduces coronary blood flow to a certain extent. Additionally, coronary artery occlusion and spasm caused by myocardial contraction of the bridge exacerbate myocardial imbalance of supply–demand (9). Increased systolic compression of the coronary artery and a shortened diastolic coronary filling time caused by tachycardia under stress conditions also exacerbate this imbalance.

The present case had Hashimoto's thyroiditis, also known as chronic lymphocytic thyroiditis, which is an autoimmune disease, and it can cause hypothyroidism. Patients with hypothyroidism have endothelial dysfunction, increased platelet activation, and increased cardiovascular risk. Hypothyroidism leads to hypercholesterolemia, increased levels of low-density lipoprotein cholesterol, and hypertriglyceridemia, promoting the development of atherosclerosis and coronary heart disease (10, 11). Studies have shown that a large number of immune cells, including macrophages and T cells, accumulate in atherosclerotic lesions. Adhesion molecules and cytokines released by these cells further activate the immune response and participate in the formation of atherosclerosis (12–14). Many autoimmune diseases, such as rheumatoid arthritis, systemic lupus erythematosus, and antiphospholipid antibody syndrome, are associated with arteritis, accelerated progression of atherosclerosis, and increased cardiovascular risk (15). Therefore, Hashimoto's thyroiditis may be associated with autoimmunization in patients or inflammatory arteritis, thereby leading to atherosclerosis. However, this possibility needs to be confirmed by further studies.

The findings of the current case suggested that MB initiated the development of coronary atherosclerotic lesions. Our patient had abnormal thyroid function caused by Hashimoto's thyroiditis or hypothyroidism, and the TSH level was elevated. TSH is one of the hormones involved in regulating lipid metabolism. High TSH levels are a risk factor for hypercholesterolemia and hypertriglyceridemia, which have an adverse effect on the lipid profile and lead to an increase in morbidity and mortality from coronary heart disease (16). Therefore, our patient received secondary prevention therapies for coronary heart disease, such as antiplatelet, lipid-lowering/plaque-stabilizing, and heart rate-slowing therapies. He also received thyroid hormone replacement therapy for hypothyroidism. After 6 months of follow-up, the patient had a good prognosis.

Hashimoto's thyroiditis, which is an autoimmune disease, not only promotes progression of atherosclerosis in arterial segments proximal to MB but also promotes progression of atherosclerosis in the entire arterial segment. Hashimoto's thyroiditis and MB increase the risk of acute coronary syndrome, and patients with this condition and MB are prone to adverse cardiovascular events, such as acute myocardial infarction. The combination of immune responses and low thyroid function contributes to progression of coronary atherosclerosis in the entire arterial segment, with more significant progression than hypothyroidism alone.

Manifestations of MB-related myocardial ischemia are not uncommon. Patients with MB have a high risk of acute myocardial infarction or even sudden death. Findings from our case suggest that early recognition of hypothyroidism and MB is important for risk stratification and prognosis in patients with myocardial necrosis and acute coronary syndrome. This early recognition may have beneficial effects on cardiovascular outcomes in patients with hypothyroidism.

## Data Availability Statement

The original contributions presented in the study are included in the article/supplementary material, further inquiries can be directed to the corresponding author/s.

## Ethics Statement

The studies involving human participants were reviewed and approved by the Ethics Committee of Qingyuan People's Hospital, the Sixth Affiliated Hospital of Guangzhou Medical University. The patients/participants provided their written informed consent to participate in this study. Written informed consent was obtained from the individual(s) for the publication of any potentially identifiable images or data included in this article.

## Author Contributions

All authors listed have made a substantial, direct and intellectual contribution to the work, and approved it for publication.

## Conflict of Interest

The authors declare that the research was conducted in the absence of any commercial or financial relationships that could be construed as a potential conflict of interest.

## Publisher's Note

All claims expressed in this article are solely those of the authors and do not necessarily represent those of their affiliated organizations, or those of the publisher, the editors and the reviewers. Any product that may be evaluated in this article, or claim that may be made by its manufacturer, is not guaranteed or endorsed by the publisher.
